# An educational intervention to reduce pain and improve pain management for Malawian people living with HIV/AIDS and their family carers: study protocol for a randomised controlled trial

**DOI:** 10.1186/1745-6215-14-216

**Published:** 2013-07-13

**Authors:** Kennedy Nkhoma, Jane Seymour, Antony Arthur

**Affiliations:** 1 Sue Ryder Care Centre for the Study of Supportive, Palliative and End of Life Care, School of Nursing, Midwifery and Physiotherapy, University of Nottingham, Queen’s Medical Centre, Nottingham NG7 2UH, UK; 2 School of Nursing Sciences, University of East Anglia, Norwich Research Park, Norwich NR4 7TJ, UK

**Keywords:** HIV/AIDS, Trial, Pain, Carers, Educational intervention, Palliative care

## Abstract

**Background:**

Many HIV/AIDS patients experience pain often due to advanced HIV/AIDS infection and side effects of treatment. In sub-Saharan Africa, pain management for people with HIV/AIDS is suboptimal. With survival extended as a direct consequence of improved access to antiretroviral therapy, the prevalence of HIV/AIDS related pain is increasing. As most care is provided at home, the management of pain requires patient and family involvement. Pain education is an important aspect in the management of pain in HIV/AIDS patients. Studies of the effectiveness of pain education interventions for people with HIV/AIDS have been conducted almost exclusively in western countries.

**Methods/design:**

A randomised controlled trial is being conducted at the HIV and palliative care clinics of two public hospitals in Malawi. To be eligible, patient participants must have a diagnosis of HIV/AIDS (stage III or IV). Carer participants must be the individual most involved in the patient’s unpaid care. Eligible participants are randomised to either: (1) a 30-minute face-to-face educational intervention covering pain assessment and management, augmented by a leaflet and follow-up telephone call at two weeks; or (2) usual care. Those allocated to the usual care group receive the educational intervention after follow-up assessments have been conducted (wait-list control group). The primary outcome is pain severity measured by the Brief Pain Inventory. Secondary outcomes are pain interference, patient knowledge of pain management, patient quality of life, carer knowledge of pain management, caregiver motivation and carer quality of life. Follow-up assessments are conducted eight weeks after randomisation by palliative care nurses blind to allocation.

**Discussion:**

This randomised controlled trial conducted in sub-Saharan Africa among people living with HIV/AIDS and their carers will assess whether a pain education intervention is effective in reducing pain and improving pain management, quality of life and carer motivation.

**Trial registration:**

Current Controlled Trials ISRCTN72861423.

## Background

It is estimated that 34 million people were living with HIV/AIDS at the end of 2010 [1]. In 2010, there were 1.8 million deaths from AIDS, and 2.7 million people newly infected globally. In the same year, 1.4 million people commenced HIV medication, an increase of 27% in the number of people receiving treatment from the previous year. Greater access to effective treatment has led to a 19% decline in deaths among people living with HIV/AIDS between 2004 and 2009.

Sub-Saharan Africa has 10% of the world’s population, but it is home to 67% of all people living with HIV/AIDS, making it the region worst-affected by HIV/AIDS [1,2]. Antiretroviral therapy can dramatically increase survival and years of healthy life, but is unavailable in some parts of the region [2]. In 2009 in sub-Saharan Africa, 37% of the population eligible for HIV medication were treated, compared with 2% seven years earlier [3].

In Malawi the prevalence of HIV/AIDS is estimated at 11% of the population aged between 15 and 49 years, with around 920,000 people living with HIV/AIDS at the end of 2010 [1,2]. Approximately 250 people are newly infected each day, and at least 70% of Malawi's hospital beds are occupied by HIV/AIDS patients, making Malawi the 12^th^ worst-affected country with HIV/AIDS worldwide [4]. Substantial progress has been made in the provision of HIV medication. By the end of 2010, an estimated 250,000 people had commenced HIV treatment representing 52% of those in need [1]. However, due to inequities within Malawi’s health system, access to HIV medication is sub-optimal [5-8]. One initiative to help deal with the challenge of accessing HIV medication has been the involvement of nurses in the prescription and administration of medications. Trained health assistants now provide HIV counselling services to patients, and this has resulted in a greater proportion of patients starting HIV medication within three weeks of diagnosis [1].

Advanced HIV disease infection and its treatment with HIV medication are associated with physical and psychological symptoms. These require focused assessment and management using locally available resources and interventions to optimise quality of life for patients and their carers [9]. The negative impact of pain on quality of life has been documented in many studies [10,11]. Pain is a major problem for people living with HIV/AIDS [12-14]. Pain is the most frequent and main cause of psychological distress [15,16]. Experiencing pain can reduce adherence to drugs and quality of life for HIV/AIDS patients [17-21].

Inadequate pain control remains a challenge for HIV/AIDS patients and has an impact on their quality of life [19,20]. Pain is experienced throughout the disease trajectory, severity being associated with later World Health Organisation (WHO) clinical stage, [22-24] with an estimated 80% of people with advanced HIV infection experiencing severe pain [25]. Pain is also experienced due to the effects of HIV medication [26,27]. With advances being made in improving access to HIV drugs in resource poor countries, HIV patients are living longer, and, therefore, experiencing pain over a longer period [28,29]. For cost-related reasons there is rarely the opportunity for second-line antiretroviral medication to be prescribed when first-line antiretroviral therapy is poorly tolerated [30]. There is a need to provide effective interventions to HIV/AIDS patients in alleviating and managing pain. Previous trials conducted in western countries of interventions to improve medication adherence have produced conflicting results; one found evidence that medication adherence and knowledge can be improved [31] and another suggested that quality of life outcomes were worse in the intervention group [32]. In a trial of a symptom management manual for people with HIV/AIDS, symptom frequency was reduced but only a small number of trial centres were in sub-Saharan Africa [9]. The majority of centres were in the United States where the healthcare context is very different. None of these trials directly involved unpaid carers, a group likely to play a key role in the management of pain of those for whom they care.

### Aim

The aim of this trial is to evaluate the effect of an educational intervention for patients with HIV/AIDS and their carers. The study will test the following hypotheses:

1. Compared with usual care, patients with HIV/AIDS who receive a pain education intervention will report less severity of pain.

2. Compared with usual care, patients with HIV/AIDS who receive a pain education intervention will report less interference of pain in their daily activities.

3. Compared with usual care, patients with HIV/AIDS who receive a pain education intervention will have a greater knowledge of pain management.

4. Compared with usual care, patients with HIV/AIDS who receive a pain education intervention will have a better quality of life.

5. Compared with usual care, carers of patients with HIV/AIDS who receive the pain education intervention will have greater knowledge of pain management.

6. Compared with usual care, carers of patients with HIV/AIDS who receive the pain education intervention will have greater motivation to provide care.

7. Compared with usual care, carers of patients with HIV/AIDS who receive the pain education intervention will have a better quality of life.

## Methods/design

### Overview of study design

The study is a two-centre randomised wait-list controlled trial. Participants (patients with HIV/AIDS and their carers) randomly allocated to a pain education intervention group receive a leaflet-based educational intervention and verbal instructions for approximately 30 minutes on pain assessment and management in addition to usual care. Participants randomly allocated to the usual care group receive standard care, but receive the leaflet-based educational intervention on completion of follow-up measures for both treatment groups (wait-list control). Participants are assessed at baseline after providing informed consent and then randomly allocated to either the pain education intervention or usual care arm of the trial. Follow-up assessments are conducted after eight weeks.

### Setting

The trial setting is that of HIV and palliative care clinics within two public hospitals in northern Malawi. Both hospitals (Ekwendeni and Mzuzu Central) provide in-patient, clinic-based and home-based care for people with HIV/AIDS that includes active treatment and palliative care. Ekwendeni Hospital provides services funded by the government. It was one of the first hospitals in Malawi to provide free HIV/AIDS medication. Mzuzu Central Hospital is government-funded and the largest referral hospital in north Malawi for people with HIV/AIDS. The population served by these hospitals includes people from both rural and urban areas.

### Study participants

Participants are people living with HIV/AIDS and their carers. All participants need to be able to read and write in English or Tumbuka (the vernacular language used in the northern part of Malawi). They must be adults aged 18 years or over.

#### Inclusion criteria for people living with HIV/AIDS

To be eligible for the trial, participants must have received a diagnosis of HIV/AIDS. Participants with other conditions, such as cancer and tuberculosis, are included if these conditions present alongside a diagnosis of HIV/AIDS. Eligible participants with HIV/AIDS must be at WHO clinical stages III or IV of HIV/AIDS, or with a CD4 cell count of less than 350 cells, when the presence of pain and other symptoms are more likely due to opportunistic infections or side effects of HIV treatment. Staging for trial eligibility is assessed from the medical records if recorded or through assessment by clinic staff if this information is not available.

#### Inclusion criteria for carers

To be eligible for inclusion, carers must be living with the person with HIV/AIDS and be identified as the individual most involved in their care.

#### Exclusion criteria for people living with HIV/AIDS

People living with HIV/AIDS will be excluded if they have health problems that may hinder cognition and communication, such as HIV-associated dementia. This is assessed by the attending clinical officer during history-taking at the initial assessment or at clinic review.

### Recruitment

People living with HIV/AIDS in Malawi typically visit the hospital (palliative care clinics and HIV clinics) with their family members. Posters about the study entitled ‘Pain Education Study’ are prominently displayed and potential participants have the opportunity to be given further information about the study directly from KN or from clinic staff.

**Figure 1:**
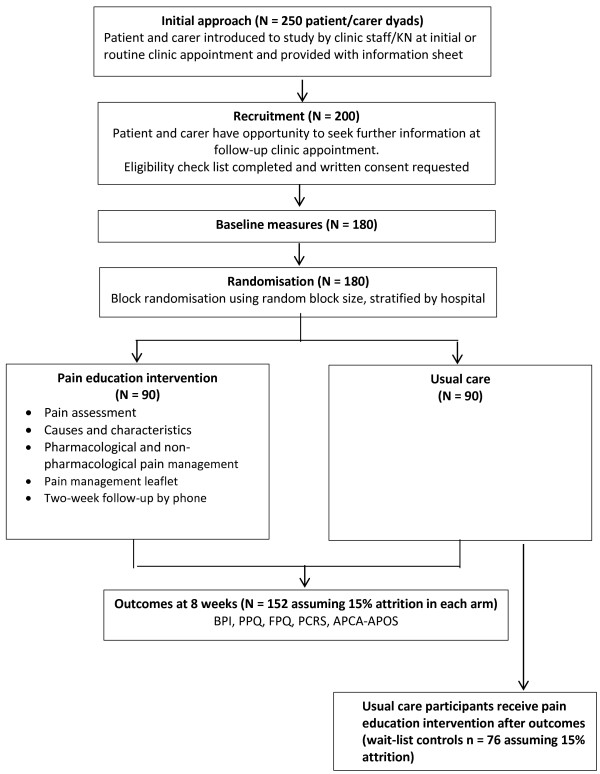
Flow diagram of study design.

The study is introduced either during the first appointment at the HIV clinic for newly registered patients or during routine appointments at the HIV clinics or palliative care clinics for those who are already receiving HIV medication (see Figure 1). KN or the staff in these clinics inform patients about the study and provide them with information sheets. Potential participants are encouraged by KN or the clinic staff to discuss with family members before making a decision to take part.

Potential participants have between two and four weeks to consider taking part in the study. During their next appointment those who are interested in taking part in the study are asked to provide written informed consent by KN. A checklist is administered to confirm that all criteria for study eligibility are met.

### Randomisation

After baseline assessments, participants are randomly allocated to the pain education intervention group or usual care group. Randomisation is implemented by KN using opaque, sealed and numbered envelopes. The envelope is opened in the presence of the participants after baseline assessments. Participants have a 50% chance of being allocated to either the pain education intervention group or usual care group. In order to limit imbalance between the treatment groups, participants are randomly assigned with block randomisation using the ‘ralloc’ command in Stata version 12 [33] Name of manufacturer: StataCorp, College Station, Texas, USA. Randomisation is stratified by the recruiting hospital. KN is not involved in the preparation of the envelopes and is blind to block size.

### Interventions

#### Usual care

Assistance with pain management for patients with HIV/AIDS is currently provided by hospital-based palliative care nurses and typically delivered in either a palliative care clinic or HIV clinic. Information relating to pain medication is typically responsive rather than proactive and *ad hoc* rather than systematic. Information is provided when requested by patients or carers. The focus is mostly restricted to pharmacological treatment of pain. Pain assessments are not usually conducted in a systematic way and not recorded routinely. It is unusual for this information to be shared with patients and/or their carers.

#### Pain education intervention

The pain education intervention is informed by a biopsychosocial approach [34] to management of pain among people with HIV/AIDS. This conceptual framework has guided the development of the intervention in targeting adequate and effective use of analgesia (biological), providing support and knowledge to minimise distress associated with poorly controlled pain (psychological), and targeting the intervention at the level of the patient/carer dyad (social). The intervention consists of a leaflet and health education session delivered face-to-face by KN to the participants at the HIV clinic or palliative care clinic. The face-to-face session takes approximately 30 minutes wherein KN explains the intervention to the patient and carer and both are given a copy of the leaflet and allowed to browse through it briefly. KN then discusses the contents of the leaflet with the participants and they are both encouraged to ask questions. After two weeks, participants receive a phone call reminder to enquire whether they have any further questions after reading the leaflet. The details of the session are reported in Table 1.

**Table 1:** Components of the pain education intervention

**Topics to be covered**	**Content**
Introductions	Participants (patient and carer) welcomed
	Introductions and clarifications as required
	Leaflet provided and participants given time to read through
Overview of pain in HIV/AIDS	Pain defined in relation to HIV/AIDS
	Possible causes of pain in HIV/AIDS discussed
	Characteristics of pain relating to HIV/AIDS
Beliefs and myths about pain in HIV/AIDS	Participants given opportunity to share beliefs about pain in relation to HIV/AIDS
	Where appropriate misconceptions dispelled
Beliefs and myths about pain medication	Ask the participants’ beliefs about use of pain medication
	Summarise and dispel misconceptions as required about pain medication
Assessment of pain in HIV/AIDS	Demonstrate with the help of body diagrams how to locate and describe pain
	Demonstrate use of pain assessment tools to rate and record pain
	Demonstrate with pain diagrams how to classify pain
	Explore type of pain experienced and strategies used to manage pain
	Discuss ways in which pain may be managed more effectively
Pharmacological management of pain	Demonstrate, using the WHO analgesic ladder, how pain is managed with medications
	Give examples of available drugs used on the WHO ladder
	Discuss most effective timing of pain medication
Non-pharmacological management of pain	Identify what non-pharmacological interventions participants are aware of and use
	Practical demonstrations on use of non-pharmacological interventions as appropriate
Other items to be covered	Participants given further opportunity to clarify any of the points discussed
	Participants encouraged to re-read the leaflet after the end of the face-to-face meeting and refer to it whenever the patient experiences pain
	Advise participants to ask for clarification about the leaflet and its contents by sending a missed call to KN who will then return the call
	Routine follow-up call at two weeks

### Measures

#### Baseline

After recruitment and obtaining written consent from participants, but prior to randomisation, baseline assessments are conducted by KN. Baseline assessments include relevant details from medical notes (date of diagnosis, current treatments) and demographics. Other measures taken at baseline are those used as outcomes for the trial.

The primary outcome is pain severity measured using the Brief Pain Inventory [35]. A range of secondary outcomes have been chosen due to the complex nature of the intervention. The time point between delivery of the intervention and follow-up assessments was chosen to be consistent with other studies of pain education [36,37]. Patients are assessed in terms of pain severity, pain interference with daily activities, knowledge of pain management, and quality of life. Carers are assessed in terms of knowledge of pain management, caregiver motivation and quality of life. These are measured as follows:

1. Pain severity is measured using the single item of the Brief Pain Inventory (BPI-PS) [35] where patients are asked to rate the severity of their pain on average over the last week. A rating is made on a 0 to 10 point scale with higher scores indicating greater severity of pain. This is consistent with the measurement of pain severity in a number of clinical trials [38]. The BPI has been used with patients with cancer and other chronic illnesses such as HIV/AIDS [15,39] and to study the management of pain in South Africa [40].

2. Pain interference with daily activities is measured using the mean score of the seven pain interference items of the Brief Pain Inventory (BPI-PI). These items measure, on a scale of 0 to 10, the degree to which the patient reports pain interfering with each of seven activities (general activity, walking, work, mood, enjoyment of life, relations with others and sleep) and is the recommended method of assessment of pain-related functional impairment in clinical trials [41].

3. For patients, knowledge of pain management is measured using the knowledge subscale of the Patient Pain Questionnaire (PPQ-K) [42]. The PPQ-K is made up of nine items asking the patient to disagree or agree with statements about the effectiveness, timing of pain medication dosage, and adequacy of pain medication dosage. Agreement/disagreement is rated on a scale of 0 to 10. Scores range from 0 to 90 with higher scores indicating greater patient knowledge of pain management.

4. For patients, quality of life is measured using the APCA African POS [43]. The APCA African POS consists of seven items directed at patients addressing pain and symptom assessment, psychological and emotional concerns. Possible scores range from 0 to 35 with higher scores indicating worse outcomes/quality of life. The tool has been developed and tested in three African countries [44].

5. For carers, knowledge of pain management is measured using the knowledge subscale of the Family Pain Questionnaire (FPQ-K) [45]. Like the PPQ-K, the FPQ-K is made up of nine items asking the carer to disagree or agree with statements about the effectiveness, timing of pain medication dosage, and adequacy of pain medication dosage. Agreement/disagreement is rated on a scale of 0 to 10. Scores range from 0 to 90 with higher scores indicating greater carer knowledge of pain management.

6. Carer motivation is measured using the Picot Caregiver Rewards Scale (PCRS) [46]. The PCRS is a 16-item scale measuring the positive consequences of caregiving. Respondents rate the degree to which items describe positive consequences of their caregiving on a 5-point Likert scale. Possible scores range from 0 to 64 with higher scores indicating more positive caregiving experience.

7. For carers, quality of life is measured using the APCA African POS [43]. The APCA African POS consists of three items directed at carers addressing the adequacy of information the family has received, confidence in caring, and level of worry. Possible scores range from 0 to 15 with higher scores indicating worse outcomes/quality of life.

While the BPI [40] and APCA African POS [43] have both been used previously in Sub-Saharan African Populations, use of the PPQ, FPQ and PCRS has been restricted to populations in western countries. Our experience immediately prior to trial recruitment of piloting these scales as part of the questionnaires among 10 patients and 10 carers suggests that they are acceptable to and understood by members of the population of patients and carers from which our sample is being recruited.

#### Follow-up

Follow-up measures are conducted after eight weeks following delivery of the intervention. Nurses blind to treatment group conduct the follow-up assessments. This is implemented during the routine appointments to the HIV or palliative care clinic.

### Sample size

We wish to be able to detect a mean difference of 10% between the treatment groups in the primary outcome measure (average pain severity in the BPI). A 10% improvement is the lower limit of changes considered clinically important [47]. Using a *P*-value cut-off of 0.05 to determine a statistically significant result, 76 people per arm of the trial will be needed to complete the study to give 80% power to detect such a difference. This is based on a review [48] that suggests that education-based interventions are able to produce this level of improvement in pain reduction, and that a standard deviation of 2.2 points is a liberal estimate of variability. To allow for 15% attrition, we will attempt to recruit 180 participants to the trial.

### Statistical analysis

We will provide a descriptive account of the two treatment groups at baseline in terms of demographics, recruiting centre, stage of HIV/AIDS and baseline values of all study outcomes. All patients and carers will be analysed according to the group to which they were randomised. Treatment groups will be compared in terms of our primary outcome measure (pain severity using the BPI-PS treated as a continuous measure) using a linear regression model with baseline BPI and treatment group and recruiting centre as covariates. Analysis of each of the six secondary outcomes (BPI-PI, PPQ-K, APCA African POS patient score, FPQ-K, PCRS, APCA African POS carer score) will be conducted using six equivalent models with estimates of treatment effect conditional on the value of the outcome at baseline. Sensitivity analysis will be reported and performed as follows: we will conduct secondary analyses that (1) adjust for variables that are potential predictors of outcome (for example, age, gender, stage of HIV/AIDS, medication use at baseline) and (2) make worst-case and best-case scenario assumptions about participants lost to follow-up using the Stata command ‘rctmiss’ [49]. Analysis will be conducted using Stata version 12 [33].

### Ethical approval

The study has been approved by the University of Nottingham Medical School Research Ethics Committee (SNMP 11042012) and National Health Sciences Research Committee of Malawi (NHSRC 1023).

## Discussion

Findings from this trial will inform the management of pain experienced by people living with HIV/AIDS. Previous trials of interventions designed to enhance self-management for people living with HIV/AIDS have been conducted either exclusively [31,32,50] or predominantly [9] in western countries. Differences in terms of culture and healthcare systems mean it is unwise to uncritically apply evidence for non-pharmacological interventions from resource rich countries to those that are resource poor. Our trial also differs from these studies in intervening at the level of the patient/carer dyad. Family carers are a crucial component in the delivery of care for people living with HIV/AIDS in Malawi and other similar African countries. Most pain management educational intervention studies have been conducted in cancer populations [36,51-53]. Our intervention is targeted at pain experienced by people living with HIV/AIDS. The intervention is simple and fits within a model of care where most healthcare contact is between patients and nurses and is supported by trained health assistants.

## Trial status

The trial commenced recruiting in September 2012. We anticipate reaching our recruitment target by June 2013.

## Abbreviations

AIDS: Acquired immunodeficiency syndrome; APCA: African palliative care association; ART: Antiretroviral therapy; BPI: Brief pain inventory; FPQ: Family pain questionnaire; PCRS: Picot caregiver rewards scale; POS: Palliative care outcomes scale; PPQ: Patient pain questionnaire; RCT: Randomised controlled trial; UNAIDS: United Nations Program of HIV and AIDS; WHO: World Health Organisation.

## Competing interests

The authors declare that they have no competing interests.

## Authors’ contributions

KN, JS and AA were responsible for the development and refinement of the protocol. KN is the principal investigator and trial manager. KN will conduct data analysis under the supervision of AA. KN and AA wrote the initial draft of the manuscript. KN, JS and AA contributed to, edited and approved the final manuscript.
